# Replication and replicability as epistemic opportunity for the humanities: reflections on a Rembrandt attribution case study

**DOI:** 10.1057/s41599-026-08046-6

**Published:** 2026-07-01

**Authors:** Charlotte C. S. Rulkens, Rik Peels, Maartje Stols-Witlox, Lex Bouter

**Affiliations:** 1 Vrije Universiteit Amsterdam, Amsterdam, Netherlands; 2 University of Amsterdam, Amsterdam, Netherlands; 3 Vrije Universiteit Amsterdam and the Amsterdam University Medical Centers, Amsterdam, Netherlands

**Keywords:** Cultural and media studies, History, Philosophy

## Abstract

Motivated by the ‘replication crisis’ that deeply impacted the biomedical, natural, and social sciences, we conducted a case study to examine the strengths and limitations of replication in the humanities. Specifically, we replicated an art historical Rembrandt attribution study. In this article we place the methodology and outcomes of the Rembrandt replication within the broader debate on replication in the humanities, with the aim of delving deeper into the epistemological implications arising from this case study in replication: What epistemic aims did the Rembrandt replication study fulfil in comparison with ‘regular’ art historical attributions? And what does this case demonstrate about the conditions under which replication and replicability are desirable and attainable in the humanities? We show that replication can serve epistemic aims beyond those of the initial study and beyond mere corroboration of findings, such as historiographical insight and methodological refinement. Replication thus operates not only as a means of confirmation but as a generative process that can advance research. We furthermore propose that replicability, understood as a context-dependent epistemic value rather than a universal standard, can function as a forward-looking strategy supporting transparency, methodological reflexivity, and cumulative knowledge-building in the humanities as well. While replication and replicability may have practical and conceptual limitations, we should take care not to discard the valuable elements they offer. Conducting replications and striving for replicability, where feasible, can complement existing practices and thus is an epistemic opportunity that deserves further exploration across a broad spectrum of humanities studies.

## Introduction

Replication studies are commonly understood as repetitions of an earlier study answering the same research question using the same or similar methods under the same or similar circumstances. They are traditionally carried out in the biomedical, natural, and social sciences to see whether the initial findings can be corroborated, and are not frequently employed in the humanities.^1^ Recently, however, discussions on the possibility and desirability of replication in the humanities (Peels and Bouter 2018a) sparked the empirical exploration of replication in the humanities by conducting a replication of an art historical Rembrandt attribution study (Rulkens et al. 2022; Rulkens et al. 2025a; Rulkens et al. 2025b).^2^ We conducted two forms of replication: first a reproduction – a repetition by the reanalysis of the data sets of the initial study with the same study protocol. And second, a conceptual replication - a repetition using an improved study protocol including new technical research methods. In this article we place the methodology and outcomes of the Rembrandt reproduction and conceptual replication within the broader debate on replication in the humanities, with the aim to delve deeper into the epistemological implications arising from this case study in replication. What epistemic aims did the Rembrandt reproduction and conceptual replication fulfil in comparison with ‘regular’ art historical attributions? And what does this case demonstrate about the conditions under which replication and replicability are desirable and attainable in the humanities? To address these questions, it is important to highlight our distinction between *replication* (the act of redoing a study, in our case a reproduction and conceptual replication) and *replicability* (the degree to which the available information on a study enables such redoing).

Our replication of an art historical study is rooted in developments in the early 2010s. At the time, various publications revealed that a large number of studies in the biomedical (Begley and Ellis 2012) and social sciences (Pashler and Open Science Collaboration 2015; Wagenmakers 2012), when repeated, did not yield sufficiently similar findings. Since replication is often seen as a hallmark of science, these differences led to a wave of doubts about the methodology and trustworthiness of the scientific studies involved and the validity of their results. Additional research revealed the wide scope of this so-called ‘replication crisis’, and surveys amongst researchers demonstrated that its high prevalence was recognized across multiple scientific disciplines (e.g., Baker 2016). Scholars list a number of causes for this crisis, including questionable research practices, publication bias, insufficient transparency, and inadequate methodology or reporting (Munafò et al. 2014; Nosek and Errington 2017). A recent scoping review reported a low replication success rate (just over 50%) across various science disciplines (Cobey et al. 2023). However, other scholars have argued that claims about replication rates, and crisis narratives themselves require careful methodological scrutiny (Devezer and Bak-Coleman 2024). Some have reframed the crisis into a ‘credibility revolution’, referring to the many effective research reforms introduced since the start of the discussion (Vazire 2018). Others advocated for a nuanced narrative and a more local assessment of the issue, as its severity and impact seem to differ substantially between disciplines (Fanelli 2018; Guttinger 2020).

In 2018, the replication debate entered a new domain, as Peels and Bouter (2018a; 2018b; 2018c) proposed that humanities studies can benefit from replication as well.^3^ In response, others disputed the relevance of replication for the humanities, providing various arguments. For one thing, they argued that research in the humanities is conducted based on different quality criteria than the sciences and that in research in the humanities multiple answers may be accurate at the same time (De Rijcke and Penders 2018). Furthermore, concerns were raised that calls for replication would be harmful for those humanities studies depending on diverse arguments and perspectives (Holbrook et al. 2019). Additionally, it was proposed that demanding replicability might induce the repudiation of epistemic diversity. By extension, this would lead to an inappropriate application of policies based on core values of the natural sciences to humanities research, discrediting and damaging humanities’ unique approach and contributions to science (Penders et al. 2019). In response, Peels et al. (2019) and Peels (2019) argued that replication is possible and desirable for at least those areas of humanities research that draw conclusions based on the collection of empirical evidence.

Brooke (2024) has argued that replication in a broader sense is inherent to all scholarly research, as all researchers revisit and build upon the findings of previous research. It is important to note that this broader notion of replication differs from the formal replication discussed in this article. Yet Brooke’s observation gives further reason to investigate the value of explicating and systematizing the replicative character of scholarly research. This systematic and more formal practice of replication is new to art history.

The value of formal replication seemed particularly relevant to examine in art historical attribution because attribution questions often carry considerable scholarly, cultural, and financial stakes. Moreover, attribution research brings together different kinds of methods and evidence: technical approaches, such as imaging and material analysis, which are closer to fields that are already concerned with replication, and more humanities-based approaches, such as visual analysis, connoisseurial judgement, and historical interpretation. This combination makes attribution a productive context for exploring how different methods may triangulate findings, while also revealing both the possibilities and limits of replication in art history.

As such, art historical attribution serves as the empirical case through which broader questions on the value of replication and replicability for the humanities are examined. In doing so, we first address the terminology of replication and replicability, as well as the theoretical implications for when findings are corroborated through replication or not. We then introduce the Rembrandt attribution case and the two forms of replication carried out: reproduction and conceptual replication. The subsequent sections use this case to analyse the existing debates on the value of replication in the humanities, and the additional epistemic aims and values that replication may serve. We then return to the broader humanities context by considering means to assess when replication and replicability may be desirable, possible, and epistemically productive.

## A pandemonium of terms

The debate around replication is complicated by the pandemonium of terms that are used. The meanings that are attached to various notions often differ, both in the broader debate as well as within disciplines. Various terms and classifications of forms of replication are used for different purposes and emphases (see, e.g. Matarese 2022; Penders et al. 2019; Fidler and Wilcox 2021; Vachon et al. 2020). It is beyond the scope of this article to provide an exhaustive overview of the different terms utilized, but it underscores the importance of explaining the definitions employed in the current study. Our understanding of these terms, as used in our Rembrandt replication study and in the current article, is based on Peels and Bouter’s earlier conceptualization (2018c). They define a *replication study* as an ‘independent repetition of an earlier study, answering the same study question by using the same or similar methods under the same or similar circumstances.’ ‘*Replication* means that a study is actually replicated, with or without reaching the same conclusions’ [italics added]. According to their definitions, a replication can be considered (un)successful depending on the similarity between the findings of the replication study and those of the initial study. When successful, the findings of the replication study corroborate those of the initial study.

We use the term *replicability* also in the manner of Peels and Bouter (2018c): ‘*replicability* [of a study] means that a study can be repeated because a detailed study methods description is available’. The term *reproducibility* that is also often used by others has in our interpretation a slightly different connotation. It indicates a minimum standard, that consists of making available all the information others would need to be able to *retrace* the line of thought of the initial research, not necessarily to actually *repeat* a study. This retracing of the line of thought of the initial research, is what two researchers of replication in the field of history, Huijnen and Huistra (2022), refer to as ‘minimal reproduction’. This involves systematically tracing back the steps of the initial researchers, starting with their conclusions. Working the other way around, so starting with the research question and ending with the conclusion, would be a ‘maximal reproduction’ according to them. We will return to this distinction by Huijnen and Huistra (2022) later in our article.

In this study, we distinguish between three forms of replication: *reproduction*, *direct replication* and *conceptual replication* (Table 1). *Reproduction* entails answering the initial study question by the *reanalysis of the data sets of the initial study* with the same study protocol. A *direct replication* entails answering the initial study question by *collection of new data* with the same study protocol. And a *conceptual replication* entails answering the initial study question by the *collection of new data with a modified study protocol* (Peels and Bouter 2018c; Peels 2019).

**Table 1:** Core terms and definitions of three forms of replication as proposed by Peels and Bouter (2018c) and Peels (2019), extended with the epistemic benefits of (un)successful replication.

Form of replication	Benefits when replication is *successful*, i.e., similar answers to the same research question	Benefits when *unsuccessful*, i.e., no similar answers to the same research question
**Reproduction:** **(Reanalysis of existing data sets with the same study protocol)**	Increase of confidence in the trustworthiness of findings	Decrease of confidence in the trustworthiness of findings Possible causes: • Deviations due to lack of control over aspects of the protocol • *And/or* differences in interpretation of the dat • *And/or* random (sampling) error • *And/or* variation in contextual factors that were not taken into account
**Direct replication: (Collection of new data with the same study protocol)**	Increase of confidence in the trustworthiness of findings	Decrease of confidence in the trustworthiness of findings Possible causes: • All of the above causes of a unsuccessful reproduction • *And/or* additional data uncovers findings that were not apparent in the initial data set
**Conceptual replication:** **(Collection of new data with a modified study protocol)**	Increase of confidence in the trustworthiness of findings *and* increase of confidence in the validity due to modifications in the study protocol (triangulation)	Decrease of confidence in the trustworthiness of findings Possible causes: • Modifications of study protocol • *And/or* differences in interpretation of the data • *And/or* random (sampling) error • *And/or* variation in contextual factors that were not taken into account • *And/or* additional data or methods uncovers findings that were not apparent in the initial data set

Before reflecting on our replication study, it is useful to consider the theoretical implications of these three forms of replication when these are successful or not. We follow Peels and Bouter (2023) in understanding successful replication as potentially increasing confidence in the trustworthiness of findings, while recognising that this relation is conditional rather than automatic. A study being replicable does not by itself make its findings trustworthy, and a successful replication does not necessarily establish their validity. Table 1 illustrates how, in theory, a successful *reproduction* increases confidence in the *trustworthiness* of the findings that were obtained based on the study design, but does not necessarily increase confidence in the *validity* of those findings. After all, one can successfully reproduce invalid results, due to repetition of the shortcomings that are part of the initial study design. When reproductions are unsuccessful, this indicates that at least one of the studies may be less trustworthy, though whether this applies to the initial study, the reproduction, or both is difficult to decide. Causes for unsuccessful reproduction can be deviations from the protocol due to lack of control over aspects of the protocol, difference in interpretation of the data, random (sampling) error, and variation in contextual factors that were not taken into account.

A successful *direct replication*, in which new data is gathered according to the same protocol, increases confidence in the trustworthiness of the findings that were obtained based on the study design, not only by corroboration itself, as was the case in the reproduction, but also by collecting new data that supports the initial findings. However, just as in the reproduction, direct replications will not necessarily increase confidence in the validity, because when the initial study design was flawed, the same flaw will perpetuate. Similarly, in the case of a reproduction, when direct replications are unsuccessful, this indicates that at least one of the studies may be less trustworthy, though whether this applies to the initial study, the reproduction, or both is difficult to decide. Causes for unsuccessful direct replication can in part be similar to those of an unsuccessful reproduction. In addition, it is also possible that additional data uncovers findings that were not apparent in the initial data set.

For a *conceptual replication* the implications are slightly different: When successful, a conceptual replication increases both the confidence in the trustworthiness of findings and their validity, as they broaden the basis on which conclusions are drawn by *triangulation*. Triangulation entails the independent confirmation of findings by applying a different approach to answer the same research question. Triangulation is applied in various fields of research, and defined by Lawlor et al. (2016) in the context of epidemiology as: ‘The practice of strengthening causal inferences by integrating results from several different approaches, where each approach has different (and assumed to be largely unrelated) key sources of potential bias.’ As conceptual replication involves the collection of new data with a modified study protocol, it is in fact a triangulation method. When unsuccessful, as in both the other categories, this indicates that at least one of the studies may be less trustworthy, though whether this applies to the initial study, the conceptual replication, or both is difficult to decide. In this case the decrease can be caused by the modifications of the study protocol, or can be due to differences in interpretation of the data. Random (sampling) error and variation in contextual factors that were not taken into account, can play a role here too. It is also possible that additional data or methods uncover findings that were not apparent in the initial data set or revealed by the initial methods.

## The Rembrandt replication

The study selected for our replication was conducted in the late 1990s and concerned the attribution of two versions of a painted portrait depicting Rembrandt, held in the collections of the Germanisches Nationalmuseum in Nuremberg and the Mauritshuis in The Hague. The initial research was disseminated by different authors for various types of different audiences.^4^ For our replication study, we relied on the publications by Buijsen (1999) and Wadum (2000), as these represent the earliest scholarly outputs of the project and provide the most detailed accounts of its methodologies, reasoning, and conclusions. Their publications were based on art historical and technical research, and included an expert meeting in which the paintings were compared in real life. It concluded that the then prevalent respective attributions of the paintings - one to Rembrandt himself and one to a student copying this work - should be reversed (Buijsen 1999; Wadum 2000).

As in the initial research, our research questions for our replication study were: Did Rembrandt not, partially or entirely paint the portraits in The Hague and Nuremberg, and how do both versions relate to each other? As mentioned above, we conducted two forms of replication: First, a reproduction, i.e., answering the initial study question by the reanalysis of the data sets of the initial study with the same study protocol. Second, a conceptual replication – a repetition using an improved study protocol including new technical research methods. This allowed us to compare the two most different forms of replication and explore their respective values. We did not perform a direct replication because having worked with the equipment similar to the original equipment earlier in our careers, we were confident that repeating the analytical techniques with this same equipment would not yield different results. In this case, the comparison between a reproduction and a conceptual replication showed more promise as it enabled us to examine the relevance and implications of these two forms of replication side by side. It also allowed us to incorporate new techniques and methods within the conceptual replication, thereby creating an opportunity to explore whether replication could contribute to methodological refinement and increased research rigor Rulkens et al. (2025a). Accordingly, our subsequent discussion focuses on the comparison between reproduction and conceptual replication. A detailed description of this replication study is provided in Rulkens et al. (2025b) and the preregistration (Rulkens et al. 2025a).

The initial study was not conducted according to a formalised, pre-determined protocol, nor did the initial publications contain methodology sections as is common in articles within the sciences (Buijsen 1999; Wadum 2000). Therefore, for the reproduction, we first needed to reconstruct the initial study protocol by assessing publications and interviewing the initial researchers. It was not possible to carry out a reproduction of all parts of the study. Among other factors, data from a case study of a group of paintings used by Wadum was partially unavailable, and including the reanalysis of all the remaining data from the case study group would have been too time-consuming for the reproduction. Furthermore, due to limited time and means, it was not possible to include an expert meeting in the reproduction. The reproduction therefore, included only those elements of the initial study that were feasible to reproduce: naked eye observation; microscopy; reanalysis of the data of the old infrared reflectography (IRR)^5^; reanalysis of the old X-Radiography (X-Ray)^6^; and reanalysis of old dendrochronology data^7^. While this restricted the extent to which the reproduction could comprehensively test the trustworthiness of the initial conclusions, the aspects we were able to replicate nevertheless yielded valuable insights. Our reflections on the epistemic aims of the reproduction later in this article should therefore be understood in light of both these constraints and the conclusions that remained possible.

The conceptual replication employed improved versions of the initial methods and introduced additional techniques. These included higher-resolution and image quality IRR; improved image-quality X-Ray; dendrochronology with a larger data set and improved technique; higher-quality digital photography including the visible and UV ranges; and new analytical techniques Hirox digital 3D-microscopy^8^ and Ma-XRF^9^. Furthermore, naked eye observations and stereo microscopy were included, and the X-sections of the initial study were re-examined.^10^ For the conceptual replication, the paintings were brought together and compared in real life during an expert meeting, as had been the case in the initial research.

During the reconstruction of the initial study, we encountered gaps in documentation, especially regarding the expert meeting and the decision-making process. In response, we developed a novel procedure: the Attribution Expert Consensus Meeting (A-ECM). The new method was introduced as part of the conceptual replication to establish replicability through formal consensus assessment and to enhance equitable knowledge exchange in attribution expert meetings. It consisted of three stages: (1) preparatory data shared with five experts and a chair; (2) a structured, one-day meeting in which experts individually assessed the paintings, completed two rounds of surveys (before and after group discussion), and participated in a final debriefing; and (3) qualitative coding of their responses to systematically examine argumentation. The A-ECM offered insight into both the convergence and divergence in expert judgement and how evidence was weighed across different participants. Both our reproduction and conceptual replication corroborated the conclusions of the initial study (Rulkens et al. 2025b).

In order to maximize the future replicability of our replication study, we published a preregistration, added addenda to that preregistration throughout the research project, and anticipated openly sharing the data gathered in the replication where possible (Rulkens et al. 2025a). Preregistration is recognized as a means to address the replication crisis in the social sciences by promoting transparency and clarifying the study design prior to data collection, thereby reducing potential biases. However, we found that its application requires careful adaptation to the study at hand. Standard preregistration templates, which are primarily developed for quantitative research, required modification to accommodate the specific features of our replication study, which integrates technical research with diverse qualitative methods (also see Rulkens et al. 2025b).

## Three forms of replication: limits in control

Although the three different forms of replication in Table 1 might seem to imply that a clear demarcation between these forms is possible, research is more unruly in practice than theory suggests. It is often not feasible to exactly repeat a study and keep every factor the same (Stroebe and Strack 2014; Lynch et al. 2015; Matarese 2022; Romero 2019), while reproductions and direct replications typically require this level of similarity. Our Rembrandt attribution case exemplified how the distinction between forms of replication may become even more complex in the context of the humanities, where the researchers’ interpretative framework plays a central role in the analysis and interpretation of data to answer the research questions. A change of researchers for a replication study, or replicating a study in a different timeframe, may therefore - in addition to changes in data or study protocols - have an impact on the findings, and by extension, the successfulness of a reproduction, direct replication or conceptual replication of a study.

Additionally, there can be practical limitations, and it might be hard to control the context of the study. In the Rembrandt case, for example, bringing together the paintings in real life may in most cases not be feasible. This stands in contrast to fields such as computational sciences, where researcher interpretation plays a much smaller role and the context of a study or conscious deviations from an initial protocol can be tightly managed, as replication is carried out through formal or algorithmic procedures.

Table 2 provides a schematic overview of these considerations. It indicates which aspects of the study can typically be controlled by replication researchers (orange), and which aspects are more difficult or impossible to control (blue).

**Table 2:** Aspects of the Rembrandt study that are within the control of replication researchers (orange) and those that are beyond their control and/or inevitably subject to change (blue).

Aspect		
**Object of study**		The study object itself is subject to plasticity and historicity: artworks may change over time due to aging, restoration, or environmental conditions, and may become unavailable
**Research team/ expertise**	Careful documentation of procedures and decision-making can help future researchers understand the process	The interpretive frameworks, expertise, and subjective judgment of the initial researchers cannot be replicated, and are not fully transparent or controllable in the replication either
**Sources**	Secondary literature is usually accessible and reusable	Direct access to technical reports and archival materials may be restricted, or in some cases lost, destroyed, or otherwise inaccessible
**Methods**	If protocols are well documented, technical procedures can be repeated or updated with improved equipment	Gaps in documentation, informal practices, or undocumented decision-making processes may prevent full replication. Additionally, access to technical methods and related required expertise may be limited
**Data sets**	Re-analysis of archived technical data (e.g., IRR, X-radiography, dendrochronology) is possible when preserved and accessible	Initial data sets may be incomplete, lost, or generated with old technologies, limiting replicability or comparability of results
**Other factors**		External factors such as funding, institutional priorities, and the possibility of bringing artworks together are often outside researchers’ control

Because of these limits of control and feasibility, the distinctions between the three forms of replication presented by Peels and Bouter (2018c) are often a matter of degree (as is the distinction between a conceptual replication and a new independent study). As a result, the implications for (un)successful replication described in Table 1, may also be subject to gradation.^11^ Moreover, the table highlights a nuance in the use of the term *replicability*: a study may in fact be sufficiently described to repeat it, but it may still be practically impossible to do so, and for this reason not be ‘replicable’.

## Minimalism and maximalism

In line with the above considerations regarding the operationalization of different forms of replication and their definitions, it is relevant to reflect on earlier systematic replication attempts in the humanities that we are aware of.^12^ A notable example is a set of three small-scale case studies in the field of history, in which students replicated articles from respectively digital history, cultural history, and economic history. The replications were supervised by Huijnen and Huistra, who subsequently published the results (Huijnen and Huistra 2022). They introduce a distinction between ‘minimal reproduction’, which entails retracing the steps of the initial researcher beginning from their conclusions, and ‘maximal reproduction’, which reverses the process by starting from the initial research questions and proceeding independently. In minimal reproduction, the aim is not to determine whether the same research process would yield the same result, but to assess whether the initial conclusions are adequately supported by the sources and methods employed. Huijnen and Huistra (2022) argue that minimal reproduction also serves a function of quality control, in addition to what they call ‘epistemic consolidation’.

They advocate this approach for history, a domain in which multiple interpretations may coexist without contradiction, a characteristic identified by De Rijcke and Penders (2018) as common to the humanities more broadly. According to Huijnen and Huistra (2022), this implies that the potential for replication in historical research is inherently limited to reconstructing the initial pathway (i.e., minimal reproduction). Therefore, they are sceptical of the applicability of ‘maximal reproduction’ in the humanities and, by extension, of the replication proposed by Peels and Bouter (2018c), which was applied in our Rembrandt case study. This is because replication, they allege, would only allow for corroboration of trivial facts, and would not add to our knowledge of the more complex issues still subject to discussion. However, they do not clarify whether - and if so, how - minimal reproduction would be able to do this. Moreover, although undisputedly valuable, the question is how novel minimal reproduction actually is, as it can be seen as an intensified form of peer review.

By implementing such a ‘maximalist’ approach in the Rembrandt case, we showed that such an approach is possible and may have substantial value, both in the reproduction and the conceptual replication. The notion that this may result in differences not merely in argumentation but also in findings, we do not consider as a limitation but rather as an asset. After all, the fact that in some cases there may be multiple and equally valid argumentations or results, does not imply that just *any* argumentation or result is valid. Maximalist replication can serve to identify incomplete or unreliably built lines of argumentation and results, *or* to increase the evidence for a certain claim by adding perspectives from a larger group of researchers, as was the case for our Rembrandt replication (Rulkens et al. 2025b). Of course, as our research question was trichotomous - e.g., was the Nuremberg painting not, partially or entirely painted by Rembrandt? It remains to be seen whether and if so, how this approach would lend itself to more multifaceted research questions, which is an issue that merits further exploration.

Finally, for art history (and for the art market, for that matter), the example of corroborating a Rembrandt attribution is not trivial, nor is its ‘epistemic consolidation’. A painting considered to be entirely painted by Rembrandt himself has a considerably higher monetary value and a fairly certain (de)attribution tells us more about the working process of the painter, and their studio practice. Those kinds of findings are important building blocks for further research into an artist, their oeuvre, the networks that they were part of, and the context in which they worked. Therefore, they are of academic importance.

## Expanding epistemic aims and values

As previously noted, the systematic and more formalized practice of replication represents a novel development within the field of art history. We now have discussed a conceptual framework concerning various forms of replication, and examined the methodology and outcomes of the Rembrandt replication alongside broader debates on replication in the humanities. This culminates in the following question: what epistemic aims did the Rembrandt reproduction and conceptual replication meet in comparison with ‘regular’ art historical attributions? Simply put: how is a replication study like this actually useful?

To address this, we must first clarify what we mean by epistemic aims and epistemic values. The literature reveals ambiguity in how these concepts are defined (Lusk and Elliott 2024; Elliott and Korf 2024). In order to avoid unclarity, we therefore provide our own definitions here. We distinguish between *specific* and *broad epistemic aims*. Specific epistemic aims refer to narrowly focused goals related to the knowledge production in a particular study. In art historical attributions, these may include distinguishing workshop practices from individual authorship, situating a work chronologically within an oeuvre, testing demarcation of an oeuvre by comparison with comparative oeuvres, clarifying provenance, and reassessing attribution decisions made by earlier scholars. Broad epistemic aims, however, refer to the overarching scholarly goals such as truth, explanation, or the development of historical, social, or cultural understanding.

We understand *epistemic values* as normative criteria used to evaluate the quality and justification of knowledge claims. They articulate desiderata that contribute to the likelihood that a proposition (claim, hypothesis, result, conclusion, theory) actually embodies knowledge, however in practice often remain implicit. In art historical attribution, epistemic values include accuracy (the degree to which an attribution corresponds to the evidence), coherence (its fit with established knowledge), explanatory power (its capacity to account for puzzling features of an artwork), transparency (the openness of methods and criteria used) and objectivity (the mitigation of biases in judgment).^13^

Since a replication entails a systematic repetition of an initial study, it can in principle fulfil the same epistemic aims and values as a regular attribution study. However, our Rembrandt reproduction and conceptual replication addressed additional epistemic aims, extending beyond assessing trustworthiness or validity of findings. They furthermore enhanced interrelated epistemic values not typically emphasized in regular attribution studies. Table 3 presents activities that were part of the reproduction and conceptual replication, together with their respective additional broad and specific epistemic aims and values. Direct replication is not included in this table, as it was not part of our case study.

**Table 3:** Activities related to the Rembrandt reproduction and conceptual replication and their related epistemic aims and values.

Form of replication	Activity	Additional specific epistemic aims	Additional broad epistemic aims	Additional epistemic values
**Reproduction:** **(Reanalysis of existing data sets with the same study protocol)**	Reconstructing the initial study protocol	Historiographical insight into the initial study	Achieving inter-subjective justification Enhancing trustworthiness; Improving methodological standards	Replicability; Equitable knowledge exchange; Enhancing values of transparency, intersubjective scrutiny and accuracy
		Identification of parts of initial study for which methodologies or course of events were unclear, informing setup conceptual replication		
	Partially reproducing a study	Assessment of the trustworthiness of findings of the reproduced elements of the initial study		
**Conceptual replication:(Collection of new data with a modified study protocol)**	Adjustment of study protocol	Improvement of the study protocol (e.g., its transparency, its replicability)		
		Inclusion of new sources, new data, new/ improved methods (both data collection methods and data analysis methods)		
	Conceptually replicating a study	Assessment of the trustworthiness of findings of the full initial study		
		Triangulation and assessment of the validity of findings of the full initial study		
**Combination: Reproduction and conceptual replication**	Comparison of findings of elements of the reproduction and the conceptual replication	Assessment of the impact of improved technologies and methods on findings of the initial study versus the conceptual replication		

In the reproduction, additional *specific epistemic aims* included gaining historiographical insight into the initial study and identifying parts of its methodology that were too opaque for a straightforward reproduction. These insights informed the design of the subsequent conceptual replication. The reproduction also allowed for partial assessment of the trustworthiness of specific elements of the initial study (for example, through reproducing dendrochronological analysis).

In the conceptual replication, additional *specific epistemic aims* included improving the initial study protocol, incorporating new sources and methods (both in data collection and analysis), introducing new data, and triangulating findings to assess the validity of the initial conclusions. It also allowed us to assess the impact of improved technologies and methods on the findings of the initial study in comparison with the technologies and methods of the conceptual replication (such as dendrochronology).

In addition to these specific aims, our case study revealed *broader epistemic aims* connected to both the reproduction and the conceptual replication. These include enhancing the trustworthiness of knowledge by testing whether other researchers can confirm the attribution, thereby increasing confidence in its trustworthiness and validity, and improving methodological standards by using replication as a testbed to reveal strengths, limitations, or ambiguities in the initial study. Such processes not only help to refine existing practices but also foster critical reflection on the methods and assumptions that underlie attribution research. Replication thus operates not only as a means of confirmation but as a generative process that advances art historical attribution research.

Building on this, the conceptual replication also introduced replicability as an *epistemic value* in attribution research. While neither replication (the act of redoing a study) nor replicability (the degree to which a study enables such redoing) is always feasible, striving for replicability where possible remains valuable. As demonstrated with the Rembrandt case, reproduction and conceptual replication can promote more robust, reflective, and transparent knowledge production, and facilitate future engagement with past research. Replicability, in this context, should not be understood narrowly as the technical possibility of repeating a study because its methods have been documented. Rather, it reflects a broader commitment to methodological refinement that enables repetition and supports interrelated epistemic values such as transparency, intersubjective scrutiny, accuracy, and equitable knowledge exchange. It is not an end in itself, but a complementary, forward-looking research orientation can strengthen the cumulative potential of attribution scholarship.

In our Rembrandt case study, we sought to enhance replicability through detailed preregistration, open sharing of data and explication of methods, and method design enabling the possibility of future repetition, such as the expert consensus procedure. Nonetheless, further work is needed to explore how replicability can be meaningfully pursued in various ways. Such efforts must take into account the diversity of research contexts, epistemic aims, and practical constraints, including available resources. Identifying strategies to support replicability that are both contextually appropriate and epistemically productive remains a key challenge for future research.

## Replication and replicability as epistemic opportunities

Our case study has demonstrated how replication in art historical attribution research can serve epistemic aims beyond those of the initial research and beyond assessing trustworthiness or validity of findings. Moreover, it has shown how the value of replicability can underpin those aims. This raises a critical question: what does this case demonstrate about the conditions under which replication and replicability are desirable and attainable in the humanities?

To work towards an answer to this question, we draw on Leonelli’s (2018) taxonomy of research contexts, which provides a helpful framework for analysing when and how replicability is achievable. She uses the term ‘reproducibility’, but her observations are equally applicable to the replicability terminology used in this article. Leonelli distinguishes between six categories of research, in the context of which replicability can encompass various things. The categories are (1) software development, (2) standardized experiments, (3) semi-standardized experiments, (4) non-standard experiments and research based on rare, unique, perishable, inaccessible materials, (5) non-experimental case description, and (6) participant observation. Penders et al. (2019), split Leonelli’s fourth category into two: ‘non-standard experiments’ and ‘research based on rare, unique, perishable, inaccessible materials,’ and include ‘immersion’ research as a category.

Our Rembrandt replication project illustrates, however, how research can span multiple categories simultaneously. For instance, certain aspects (e.g., dendrochronological analysis) are suitable to higher degrees of standardization, while others (e.g., expert connoisseurship) involve situated judgment and less controllable contexts. Recognizing this heterogeneity within disciplines and even within individual studies opens up a broader spectrum of epistemic opportunities. We would like to suggest that (replication) researchers might benefit from identifying elements of their study in relation to Leonelli’s six areas, or the eight areas as proposed by Penders et al. (2019). By looking at their studies through the lens of the above categories, both initial and replication researchers can create the conditions for replicability of (parts of their) study, or respectively, design a replication study with different kinds of replications in mind, related to a certain element of a study.

But there is an additional consideration, as Leonelli furthermore argues that replicability not only has various characteristics depending on the category, but also that it may be more or less applicable depending on (A) the degree of control over the environment of a study, and (B) the extent of dependence on statistics as an inference tool of the study. It seems to us that the Rembrandt replication illustrated that here too, there might be opportunities to increase replicability. First of all, although the environment could not be fully controlled in the Rembrandt replication, there were opportunities to adjust study methods to maximize this control and by extension the replicability of the study. For instance, as discussed earlier, by creating the A-ECM as a fixed framework within which expert meetings can be repeated and which guides and documents the scholarship process. Second, although the A-ECM did not rely on statistics as an inference tool, there was an opportunity to quantify the opinions of the experts in relation to the research question by using multiple-choice questions and qualitative coding. Clearly, this quantification cannot fully capture the complex argumentation underlying an attribution, but it can complement this argumentation and provide new ways to communicate and summarize such findings more transparently.

To claim, as we do, that replicability is an epistemic value, is, of course, to make a normative claim. We would like to emphasize, though, that it does not follow that replicability is the hallmark of true science, let alone a *conditio sine qua non* for genuine academic scholarship. We are not alone in this careful approach to replicability as an epistemic value. Treating replicability, let alone successful replication, as a universal *conditio sine qua non* for quality in science has been criticized by several authors, even for the sciences (Leonelli 2018). Matarese (2022), for instance, states that it is problematic in the first place because of the inconsistency in concepts used for replication. It therefore remains unclear which perception of replication any gold standard would entail. Moreover, our above explanations illustrate the complexity of replication studies and the diverse forms they can take. But as we demonstrated, the act of replication (in our case a reproduction and conceptual replication) can serve various epistemic aims in attribution research, and replicability as a value can underpin those aims. We should therefore not discard valuable elements of replication and replicability while addressing their limitations and possible ambiguities. Replicability is a *pro tanto value* and that value may be outweighed by other values so that all-things-considered, other values have priority, values that can even potentially clash to some degree with replicability.

Leonelli opposes the view that replication is an ideal that, even though unattainable in certain cases, remains something researchers should strive for to the fullest extent possible. She argues that this comes with the risk of idealizing replicability and harming epistemic diversity, a concern that was also raised by Penders et al. (2019) and mentioned before. We agree with these concerns, but we do think that future replicability can be aimed for without idealizing replication, without neglecting unique features or practical limitations in specific studies that make replication hard or sometimes even impossible, and without devaluing irreplicable parts or types of research. We consider it a method complementary to current practices and suggest that even if not *all* elements of a study are controllable or quantifiable, this should not exempt humanities researchers from trying to maximize transparency about their process of scholarship. Considering the replicability of humanities studies, and enhancing possible future replication as values is a powerful means towards this end.

## Conclusion

By empirically testing both a reproduction and conceptual replication of an art historical attribution study, we developed several more general ideas about replication in humanities studies. First of all, we found that preregistration can serve purposes beyond replicability: it can support methodological reflexivity and transparency, help to mitigate bias, and facilitate process management and documentation. This suggests that other humanities research projects may also benefit from context-sensitive forms of preregistration. Further research is needed to explore how these practices can be effectively developed and implemented across different types of research.

More importantly, our case study demonstrates that reproduction and conceptual replication can fulfil epistemic aims that go beyond those typically pursued in the initial research. Such additional aims are often underacknowledged in debates about replication. The epistemic advantages of reproduction and conceptual replication can be related to the past (contributing to the historiography of the study), the present (assessing trustworthiness of the findings, improvement of methodologies, and enriching existing argumentation), and the future (identifying opportunities for improving transparency and replicability). Taken together, these forms of replication can thus operate not only as a means of confirmation, but also as a generative process that can advance research.

Nevertheless, we agree with earlier authors that replication is a rich and complicated issue that may entail different things in different kinds of studies, and that it may not be possible or desirable in all situations. It should therefore not be treated as a *conditio sine qua non* for quality in research. However, while replication and replicability may have practical and conceptual limitations, we should take care not to discard the valuable elements they offer. We propose that *striving for replicability*, understood as a context-dependent epistemic value rather than a universal standard, can be a forward-looking strategy complementing existing research traditions by supporting transparency, reflexivity, and cumulative knowledge-building in the humanities as well. Additionally, looking for ways to maximize transparency of expert knowledge as we did for the Rembrandt conceptual replication, might be a way to further investigate the epistemology of connoisseurship and the role of expertise in art historical studies and humanities studies more broadly.

As argued by various authors in the context of the sciences, we encourage humanities researchers not to think of replication and replicability as a scientistic attack on the credibility of their area of research or as a method for unmasking fraudulent research. Rather, it should be understood as an opportunity to foster progress in the humanities, in a way that is sensitive to the various aims, methods, and interpretive richness of the humanities. At the same time, our observations underscore that the sciences - humanities divide, while heuristically useful and deeply embedded in institutional structures, is far from absolute. Methodological elements like standardization, transparency, and replication can be meaningfully and selectively adapted across disciplinary boundaries without compromising humanistic inquiry. Conversely, it reveals how aspects of humanistic inquiry such as expert judgment and methodological pluralism may also be present in scientific domains. In this sense, the Rembrandt replication case study highlights the shared epistemic challenges and opportunities that span disciplinary boundaries.

Replication and replicability should complement current research practices. Future research, including replication case studies exploring other areas of humanities research, may help to get a more refined understanding of the epistemic value of replication and replicability in the humanities and its various subdisciplines. However, prioritization of promising areas and research questions for conducting replication and enhancing replicability should be done locally, with the researchers themselves as experts in the epistemologies that are central to their area of research. A starting point would be for humanities researchers to consider which elements of their studies lend themselves to replication, and to become alert to the epistemic advantages of increasing their replicability accordingly. We hope that the Rembrandt replication study has opened the door to further exploration across a broader spectrum of humanities disciplines.

## Data Availability

All openly accessible data and supplementary material related to this study is stored on https://osf.io/vdkax/overview.

## Footnotes

1 While we draw on the distinction between the humanities and the sciences to frame our case study, we are aware that this binary is conceptually limited and often blurs in practice. Our use of this terminology uses the distinction as a contextual entry point and is heuristic rather than categorical.

2 In response to the debate on replication in the humanities, some of us also carried out a replication study in the field of the history of science and religion (Brooke 2024; Brown 2024; Pear et al. 2024; Peels et al. 2024b; Peels et al. 2024a; Van Eyghen et al. 2024; also Rulkens et al. 2022).

3 Although replication is uncommon in the humanities, the field of experimental philosophy – an area of research that integrates data-driven methods from the social sciences into empirical philosophy studies – has actively engaged with issues of replicability and developed practices to monitor and enhance it within their studies (see, e.g. Cova et al. 2021).

4 The other publications are: Buvelot, Quentin and Jørgen Wadum, ‘Rembrandt’s early self portrait unmasked’, *Mauritshuis in focus*, 12, 3 (1999), 32-42.; Elst, Caroline van der and Jørgen Wadum, ‘Attribution et désattribution’, *Dossier de l’art*, 61 (1999), 34-43.; Buijsen, Edwin, ‘Rembrandt’s *Self-Portrait with Gorget*: an ongoing debate’, *Oud Holland. Journal for Art of the Low Countries*, 114, 2 (2000), 155-163.; Hess, Daniel, *In Rembrandts Werkstatt: Der Meister in Original, Kopie und Studie*, ed. by Herman Maué (Nuremberg: Cicero Publishing, 2001); Suchtelen, Ariane van, ‘Portrait of Rembrandt (166-1669) with gorget’, in *Portraits in the Mauritshuis*, ed. by Quentin Buvelot (The Hague: Mauritshuis/ Zwolle: Waanders Uitgevers, 2004), pp. 225-233.

5 Infrared Reflectography (IRR) is an imaging technique that uses the difference in reflection and absorption of infrared rays by certain materials to make layers under the surface of a painting visible. It can detect underdrawings made with carbon containing (black) pigments.

6 X-radiography is an X-Ray based technique that uses the difference in resistance of lighter and heavier atoms to the penetration of radiation to make (parts of the) the structure and layers under the surface of a painting visible.

7 Dendrochronology can determine the age and provenance of oakwood by comparing the patterns of growth rings with average ring patterns in a particular region.

8 Hirox digital 3D-microscopy is a high resolution imaging method showing the topography of the surface of paintings.

9 Macro X-ray Fluorescence scanning (Ma-XRF) is an imaging technique that visualises the distribution of chemical elements of paintings.

10 X-sections, Hirox digital 3D-microscopy and digital photography in UV and RAK were only available for the MH version; X-sections were taken before but were not part of the argumentation in the initial study, therefore these are only considered within the conceptual replication.

11 As a consequence, when a study fails to replicate, it may be difficult to identify the specific reasons for the unsuccessful replication, i.e. whether it is due to substantial deviations from the initial protocol or not. Colaço et al. (2022) therefore propose that replication researchers should consult with the initial researchers to determine which deviations from the protocol are acceptable and which are not.

12 See note iii.

13 Although transparency and efforts to mitigate bias can be values in attribution research, they are not always optimally embedded in practice.

## References

[CR1] Bak-Coleman J, Devezer B (2024) Claims about scientific rigour require rigour. Nat Hum Behav 8 (1890–1891). 10.1038/s41562-024-01982-w 39317793

[CR2] Baker M (2016) 1500 scientists lift the lid on reproducibility. Nature 533(7604):452–454. 10.1038/533452a 27225100

[CR3] Begley CG, Ellis LM (2012) Raise standards for preclinical cancer research. Nature 483(7391):531–533. 10.1038/483531a 22460880

[CR4] Brooke JH (2024) On the experience of being replicated. Zygon: J Religion Scie 59(2). 10.16995/zygon.11501

[CR5] Brown J (2024) Crisis, Confidence, and the limits of replication. Zygon: J Religion Sci 59(2). 10.16995/zygon.11502

[CR6] Buijsen E (1999) Self portrait with gorget Portrait of Rembrandt with a gorget. In: White C Buvelot Q (eds) Rembrandt by himself. Royal Cabinet of Paintings Mauritshuis, The Hague, pp 112–117

[CR7] Cobey KD, Fehlmann CA, Christ Franco M, Ayala AP, Sikora L, Rice DB, Xu C, Ioannidis JP, Lalu MM, Ménard A, Neitzel A, Nguyen B, Tsertsvadze N, Moher D (2023) Epidemiological characteristics and prevalence rates of research reproducibility across disciplines: A scoping review of articles published in 2018-2019. eLife 12:e78518. 10.7554/eLife.78518 PMC10322148 37341380

[CR8] Cova F, Strickland B, Abatista A, Allard A, Andow J, Attie M, Beebe J, Berniūnas R, Boudesseul J, Colombo M, Cushman F, Diaz R, N’Djaye Nikolai van Dongen N, Dranseika V, Earp BD, Torres AG, Hannikainen I, Hernández-Conde JV, Hu W, Jaquet F, Khalifa K, Kim H, Kneer M, Knobe J, Kurthy M, Lantian A, Liao S, Machery E, Moerenhout T, Mott C, Phelan M, Phillips J, Rambharose N, Reuter K, Romero F, Sousa P, Sprenger J, Thalabard E, Tobia K, Viciana H, Wilkenfeld D, Zhou X (2021) Estimating the Reproducibility of Experimental Philosophy. RevPhilPsych 12(1):9–44. 10.1007/s13164-018-0400-9

[CR9] De Rijcke S, Penders B (2018) Resist calls for replicability in the humanities. Nature 560(7716):29–29. 10.1038/d41586-018-05845-z 30065334

[CR10] Elliott KC, Korf R (2024) Values in science: what are values, anyway?. Eur Jnl Philos Sci 14(4):53. 10.1007/s13194-024-00615-3

[CR11] Fanelli D (2018) Is science really facing a reproducibility crisis, and do we need it to? Proc Natl Acad Sci USA 115(11):2628–2631. 10.1073/pnas.1708272114 PMC5856498 29531051

[CR12] Fidler F, Wilcox J (2021) Reproducibility of Scientific Results. The Stanford Encyclopedia of Philosophy

[CR13] Guttinger S (2020) The limits of replicability. Eur Jnl Philos Sci 10(2):10. 10.1007/s13194-019-0269-1

[CR14] Holbrook JB, Penders B, de Rijcke S (2019) The humanities do not need a replication drive. In: CWTS. https://www.cwts.nl:443/blog?article=n-r2v2a4&title=the-humanities-do-not-need-a-replication-drive

[CR15] Huijnen P, Huistra P (2022) On the use of replications in history: A white paper (1.1). Zendono. 10.5281/zenodo.7037401

[CR16] Lawlor DA, Tilling K, Davey Smith G (2016) Triangulation in aetiological epidemiology. Int J Epidemiol 45(6):1866–1886. 10.1093/ije/dyw314 PMC5841843 28108528

[CR17] Leonelli S (2018) Rethinking Reproducibility as a Criterion for Research Quality. In: Fiorito L., Scheall S., Suprinyak C. E. (eds) Research in the History of Economic Thought and Methodology. Emerald Publishing Limited, pp 129–146

[CR18] Lusk G, Elliott KC (2024) Against global aims for science: values, epistemic priority, and a local aims approach. Synthese 204(2):47. 10.1007/s11229-024-04633-9

[CR19] Lynch JG, Bradlow ET, Huber JC, Lehmann DR (2015) Reflections on the replication corner: In praise of conceptual replications. Int J Res Mark 32(4):333–342. 10.1016/j.ijresmar.2015.09.006

[CR20] Matarese V (2022) Kinds of Replicability: Different Terms and Different Functions. Axiomathes 32(2):647–670. 10.1007/s10516-021-09610-2

[CR21] Munafò M, Noble S, Browne WJ, Brunner D, Button K, Ferreira J, Holmans P, Langbehn D, Lewis G, Lindquist M, Tilling K, Wagenmakers E-J, Blumenstein R (2014) Scientific rigor and the art of motorcycle maintenance. Nat Biotechnol 32(9):871–873. 10.1038/nbt.3004 25203032

[CR22] Nosek BA, Errington TM (2017) Making sense of replications. eLife 6: e23383. 10.7554/eLife.23383 PMC5245957 28100398

[CR23] Open Science Collaboration (2015) Estimating the reproducibility of psychological science. Science 349(6251):aac4716. 10.1126/science.aac4716 26315443

[CR24] Pashler H, Wagenmakers E (2012) Editors’ Introduction to the Special Section on Replicability in Psychological Science: A Crisis of Confidence?. Perspect Psychol Sci 7(6):528–530. 10.1177/1745691612465253 26168108

[CR25] Pear R, Van Den Brink G, Peels R (2024) Jewish Responses to Copernican Thought: A Conceptual Replication of John Hedley Brooke’s Chapter on Scientific and Religious Reform. Zygon: J Religion Sci 59(2). 10.16995/zygon.11499

[CR26] Peels R (2019) Replicability and replication in the humanities. Res Integr Peer Rev 4(1):2. 10.1186/s41073-018-0060-4 PMC6348612 30705761

[CR27] Peels R, Bouter L (2018a) Replication is both possible and desirable in the humanities, just as it is in the sciences - Impact of Social Sciences. In: Impact of Social Sciences - Maximizing the impact of academic research. https://blogs.lse.ac.uk/impactofsocialsciences/2018/10/01/replication-is-both-possible-and-desirable-in-the-humanities-just-as-it-is-in-the-sciences/

[CR28] Peels R, Bouter L (2018b) Humanities need a replication drive too. Nature 558(7710):372–372. 10.1038/d41586-018-05454-w 29921855

[CR29] Peels R, Bouter L (2018c) The possibility and desirability of replication in the humanities. Palgrave Commun 4(1):95. 10.1057/s41599-018-0149-x

[CR30] Peels R, Bouter L, Van Woudenberg R (2019) Do the humanities need a replication drive? A debate rages on. In: Retraction Watch. https://retractionwatch.com/2019/02/13/do-the-humanities-need-a-replication-drive-a-debate-rages-on/

[CR31] Peels R, Pear R, Van Eyghen H, Van Den Brink G (2024a) Replication in the humanities in action: reflections on a direct and a conceptual replication in the history of science and religion. Zygon: J Religion Sci 59(2). 10.16995/zygon.11500

[CR32] Peels R, Van Den Brink G, Van Eyghen H, Pear R (2024b) Introduction: Replicating John Hedley Brooke’s Work on the History of Science and Religion. Zygon: J Religion Sci 59(2). 10.16995/zygon.11255

[CR33] Peels R, Bouter L (2023) Replication and trustworthiness. Account Res 30(2):77–87. 10.1080/08989621.2021.1963708 34346793

[CR34] Penders B, Holbrook JB, De Rijcke S (2019) Rinse and Repeat: Understanding the Value of Replication across Different Ways of Knowing. Publications 7(3):52. 10.3390/publications7030052

[CR35] Romero F (2019) Philosophy of science and the replicability crisis. Philos Compass 14(11):e12633. 10.1111/phc3.12633

[CR36] Rulkens CCS, Van Eyghen H, Pear R, Bouter L, Stols-Witlox M, Van den Brink G, Meloni S, Buijsen E, Van Woudenberg R (2022) Guest Post — Exploring the Strengths and Limitations of Replication in the Humanities: Two Case Studies. https://www.cos.io/blog/exploring-the-strengths-and-limitations-of-replication-in-the-humanities. Accessed 8 Mar 2025

[CR37] Rulkens CCS, Peels R, Bouter L, Stols-Witlox M, Meloni S, Buijsen E (2025a) Replicating a Rembrandt Study. Center For Open Science. 10.17605/OSF.IO/VDKAX

[CR38] Rulkens CCS, Peels R, Stols-Witlox M, Meloni S, Lechner IM, Bouter L (2025b) The attribution of two portraits of Rembrandt revisited: A replication study in art history. Humanit Soc Sci Commun 12:1347. 10.1057/s41599-025-05523-2

[CR39] Stroebe W, Strack F (2014) The Alleged Crisis and the Illusion of Exact Replication. Perspect Psychol Sci 9(1):59–71. 10.1177/1745691613514450 26173241

[CR40] Van Eyghen H, Van Den Brink G, Peels R (2024) Brooke on the Merton thesis: a direct replication of John Hedley Brooke’s chapter on scientific and religious reform. Zygon: J Religion Sci 59(2). 10.16995/zygon.11497

[CR41] Vachon B, Curran JA, Karunananthan S, Brehaut J, Graham ID, Moher D, Sales AE, Straus SE, Fiander M, Paprica A, Grimshaw JM (2020) A concept analysis and meta-narrative review established a comprehensive theoretical definition of replication research to improve its use. J Clin Epidemiol (129):176–187. 10.1016/j.jclinepi.2020.07.006 32682961

[CR42] Vazire S (2018) Implications of the Credibility Revolution for Productivity, Creativity, and Progress. Perspect Psychol Sci 13(4):411–417. 10.1177/1745691617751884 29961410

[CR43] Wadum J (2000) Rembrandt under the Skin. The Mauritshuis “Portrait of Rembrandt with Gorget” in retrospect. Oud Holl 114(2/4):164–187

